# Convenient Formulæ

**Published:** 1861

**Authors:** 


From the *Journal of Materia Medica* we extract the following collection of convenient formulæ:

FORMULA FOR THE ADMINISTRATION OF PROPYLAMINE.

℞ Propylamini Chloridi................gr. xxxvj.
Sacchari Pul..........................ʒ ij.
Aq. Menthæ Pip....................f ℥ vj. M.
ft. solutio.

S. Dose.—A tablespoonful every two hours.

ASTRINGENT WASH FOR THE GUMS.

℞ Acid Tannici....................... 8 parts.
Alcohol (86°).....................120 "
Tinct. Benzoës.................... 8 "
Essent. Menth..................... 2 "

M. and filter. Mix a few drops of this liquid with water, and wash the gums two or three times a day.—*Dental Cosmos.*

McLEAN'S NEURALGIC LINIMENT.

℞ Extr. Belladonnæ..................4 grains.

Aq. Ammoniæ.......................6 fl. ounces.

Ol. Terbinth.......................½ "

Ol. Olivæ.........................½ "

Tinct. Opii........................2 "

Apply during the paroxysm.—*Druggist's Circular.*

IODIZED LIQUID FOR DISINFECTING WOUNDS.

Dr. Marechal, of Calve, prescribes:

Iodine..............Ffteen and a half grains.
Iodide of potassium...Thirty-one "
Distilled water.......Three pints.

Apply compresses soaked in this liquid to the wounds, changing them several times a day, or, without changing the compresses, keep them saturated with the iodized solution.—*Druggist*, from *Répert. de Pharm.*

STEARATE OF IRON IN CHANCRE.

Ricord has employed, for seven months, in the Hospital du Midi, an ointment and plaster of stearate of iron, as a dressing for soft or phagedenic chancres. They have been of great service in all cases where the phagedenic action resisted the numerous means usually employed. M. Braitte, pharmaceutist of the hospital, gives the following formulæ for these preparations:

Ointment of Stearate of Iron.—Take sulphate of iron, 500 grammes; Marseilles soap, 1000 grammes. Dissolve the sulphate of iron in about 1500 grammes of water, and the soap in an equal quantity of water. On pouring one solution into the other, a whitish green precipitate is obtained, which is dried, and then melted at a moderate temperature of 80°–84° R. Add to the melted mass, on cooling, 40 per cent. of essence of lavender, and stir it constantly until it becomes perfectly cold.

Sparadrap of Stearate of Iron—Braitte's Plaster.—Take of the stearate of iron, obtained by the process directed above, q. s.; melt it at a moderate temperature, and spread it on muslin like the ordinary sparadrap.—*Journal de Pharm.*, *Savannah Jour. Med.*

THOMPSON'S EYE-WATER.

℞ Sulphate of copper.............Ten grains.
Sulphate of zinc..............Two scruples.
Rose water....................Two pints.
Tinct. of saffron, Tinct. of camphor, each..............Half an ounce.

Mix and filter.—*Druggist's Circular.*

OINTMENT FOR WARTS.

℞ Chromate of potass...............Two grains.
Lard ...........................One drachm.

Mix. Rub the warts with it twice a day, for thsee or four weeks.—*Druggist*, from *Bull. de Therap.*

GLYCEROLE OF ZINC.

℞ Sulphate of Zinc..................Two drachms.
Glycerine......................Two ounces.

Triturate together until the sulphate is completely dissolved. A good application for external hæmorrhoids, brushed over with a camel's hair brush.—*Dr. C. A. Hartmun, Cleveland.*

DYSPEPTIC BITTERS.

℞ Gentian root, bruised............Two ounces.
Orange Peel.................. " "
Quassa, rasped..................Half ounce.
Cardamon seed, bruised,.......... " "
Caraway " .......... " "
Cloves " ...........Two drams.
Diluted alcohol.................One gallon.

Mix, and prepare according to art.—*Druggists Circular.*

### BROWN'S BRONCHIAL TROCHES.

℞ Powdered extract liquorice............1 pound.
Powdered sugar..........................1½ pounds.
Powdered cubebs.........................4 ounces.
Powdered gum arabic.....................4 ounces.
Extract of conium.......................1 ounce.

—*Druggists Circular.*

### CITRATE OINTMENT MADE WITH BEAR'S OIL.

Mr. Wm. Pr. Creecy, of Vicksburg, Miss., sent a specimen of this preparation to the editor of the *American Journal of Pharmacy*, who publishes the recipe:

Mercury...............................℥ j.

Nitric acid...........................f ʒ xiv.

Bear's oil............................f ℥ xiij.

Heat the oil in a porcelain capsule to 200 deg. F., and add the solution of nitrate of mercury gradually, stirring continuously till effervescence ceases, and frequently till cool, with a glass or porcelain spatula.

### PRESCRIPTION FOR CONSUMPTION.

Dr. C. T. Horton (*Baltimore Journal of Medicine*), uses the following prescription in the treatment of consumption, Bronchitis, etc.:

℞ Etherial tincture of Indian Hemp....½ pint.

Extract of liquorice..................½ lb. (Troy?)

Carbonate of potassa..................½ "

Warm water............................1 gallon.

The dose of this is a table-spoonful three times a day, gradually increased to two. The ethereal tincture is obtained by dissolving two ounces of the alcoholic extract of hemp in a pint of sulphuric ether.

### EMBROCATION FOR INFLAMMATION OF THE MAMMÆ.

Dr. E. M. Hale recommends the following embrocation for inflammation of the mammæ:

℞ Fluid extract of aconite............1½ ounces.
" " phytolacca..........1 ounce.
Iodine potassium......................1 dram.
Warm water............................1 pint.

Wet linen compresses and apply constantly. No application with which I am acquainted will so surely prevent tumors of the mammæ from taking on the suppurative process. Even if suppuration have taken place, it resolves the indurations, and relieves the pain.—*Med. and Surg. Reporter.*

